# A Compact Broadband Antenna with Dual-Resonance for Implantable Devices

**DOI:** 10.3390/mi10010059

**Published:** 2019-01-16

**Authors:** Rongqiang Li, Bo Li, Guohong Du, Xiaofeng Sun, Haoran Sun

**Affiliations:** College of Electronic Engineering, Chengdu University of Information Technology, Chengdu 610225, China; liyq2011@cuit.edu.cn (R.L.); throbb@163.com (B.L.); dghong@cuit.edu.cn (G.D.); sunxf@cuit.edu.cn (X.S.)

**Keywords:** implantable antenna, patch antenna, biotelemetry, specific absorption rate (SAR), Medical Device Radiocommunications Service (MedRadio) band

## Abstract

A compact broadband implantable patch antenna is designed for the field of biotelemetry and experimentally demonstrated using the Medical Device Radiocommunications Service (MedRadio) band (401–406 MHz). The proposed antenna can obtain a broad impedance bandwidth by exciting dual-resonant frequencies, and has a compact structure using bent metal radiating strips and a short strategy. The total volume of the proposed antenna, including substrate and superstrate, is about 479 mm^3^ (23 × 16.4 × 1.27 mm^3^). The measured bandwidth is 52 MHz (382–434 MHz) at a return loss of −10 dB. The resonance, radiation and specific absorption rate (SAR) performance of the antenna are examined and characterized.

## 1. Introduction

Biotelemetry provides transmission of physiological signals from inside the human body to outside it, or vice versa. One of its applications is in the field of implantable medical devices (IMDs) [1]. An implantable antenna is a key component to communicate wirelessly between external equipment and IMDs in a human body [2,3]. Usually, an implantable antenna is used in the medical implant communications service (MICS) band (402–405 MHz), which was extended to the Medical Device Radiocommunications Service (MedRadio) band (401–406 MHz) in 2009. The implantable antenna in the human body faces many challenges, such as miniaturization, bandwidth, biocompatibility, and human body safety considerations.

Planar inverted-F antennas (PIFAs) have been used in the design of implantable antennas by many research groups because of their advantages [4,5,6,7,8,9]. Compared with conventional microstrip antennas, PIFAs have some desirable features, such as smaller size and nearly omnidirectional far-field radiating patterns. On the one hand, miniaturization is a key requirement for an implantable antenna due to some strict size constraints inside the human body. Kimi et al. [4] proposed two compact implanted antennas by using meandered and spiral structures, but they both have a larger size. Liu et al. [5] realized a miniaturized implantable antenna by embedding some slots in the ground plane. However, the slotted ground designs may be limited to application scenarios where there are no conductors on the ground plane. Li et al. [6] proposed a miniaturized implantable antenna by continually meandering two radiating edges of a square ring. We proposed a compact semi-circular implantable antenna by cutting three arc-shaped slots in a semi-circular patch [7]. However, the antennas in [6,7] have narrow bandwidth. On the other hand, an implantable antenna needs a wide impedance bandwidth to avoid frequency shift due to intersubject variations of the electrical properties of human body tissues and inaccuracies of fabrication and testing. To broaden the antenna impedance bandwidth, some methods have been used. Liu et al. [8] designed an implantable antenna by arranging a rectangular three-layer slotted patch structure. However, the stacked structure usually has a large thickness. A pi-shaped PIFA structure with two meandered strips was proposed for implantable biotelemetry in [9], but this antenna has a relatively large size. In addition to planar inverted F antennas, some other technologies have also been used to design wideband implantable antennas, such as monopole antennas [10], dipole antennas [11], loop antennas [12] and slot antennas [13]. An implantable broadband antenna was designed by combining a sigma-shaped monopole radiator and a novel C-shaped antenna to excite two modes [10]. Xu et al. [11] realized the bandwidth enhancement of a conformal implantable antenna by connecting a strip and a simple dipole to obtain dual-resonance. In [12], a flexible loop antenna by introducing three complementary split ring resonators (CSRRs) was used to improve the antenna impedance matching and obtain broad bandwidth. In [13], a coplanar waveguide-fed wideband dual-ring slot antenna was proposed to improve the antenna gain. Antennas in references [11,12,13] were designed as flexible and conformal structures for some special application scenarios.

In this paper, we propose a compact broadband implantable antenna with dual resonance for implantable medical devices. The metal radiating strips of the antenna were properly bent to obtain dual-resonant frequencies and a compact structure. Details of the antenna design and experimental results are presented and discussed.

## 2. Antenna Structure and Design

### 2.1. Antenna Structure

**Figure 1:**
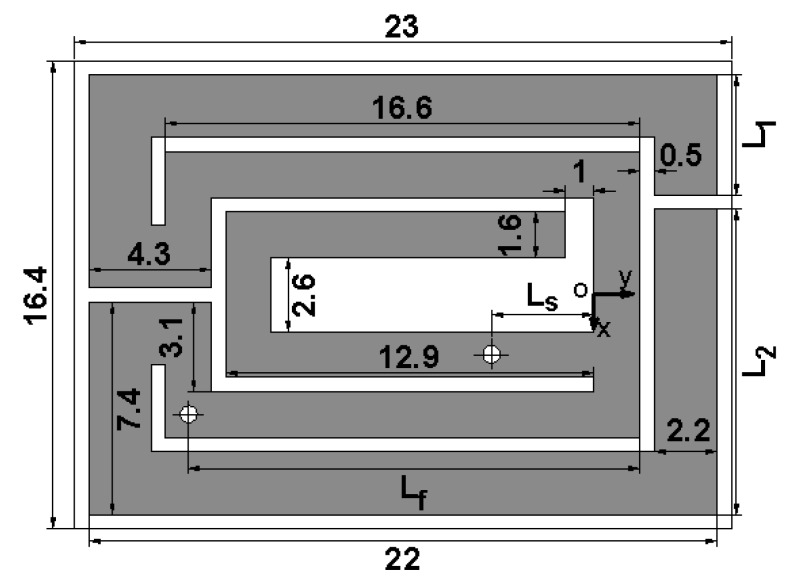
Geometry of the proposed implantable antenna (unit: mm).

**Figure 2:**
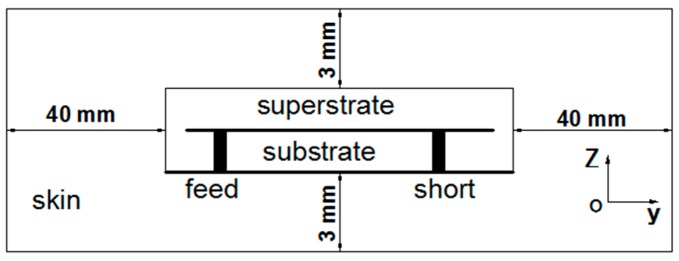
Side view of simulation model for the proposed implantable antenna.

Figure 1 illustrates the geometry of the radiating metal layer, which consists of two mutually inverted rectangular opening rings and a C-shaped strip. The C-shaped strip has a short via and is surrounded by the inner of two opening rings. Thus, the proposed antenna can form a PIFA structure and resonate at a relatively low frequency. The antenna is fed by a 50 Ω coaxial cable, which is located at the internal rectangular opening ring. Figure 2 shows the one-layer skin simulation model based on [14] for the proposed implantable antenna, placed 3 mm from the top and bottom of the skin surface, and 40 mm from the other surfaces of skin. The electrical property parameters of this skin mode at 402 MHz are *ε_γ_* = 46.7, *σ* = 0.69 s/m, in the simulation. This antenna is fabricated on a 0.635-mm-thick Rogers 6010 substrate, whose dielectric constant and loss tangent are respectively 10.2 and 0.0023, and covered by a superstrate of the same material. It is worth noting that, according to [14], the model in Figure 2 is a simplification of a three-layer model with similar return loss performance. In the three-layer model, the antenna is located under the skin adjacent to the fat layer. In reality, an antenna does not directly touch human tissue, which is placed in an implantable device, and the implantable device is placed in the human body. Some optimum parameters of the antenna derived using the high frequency structure simulator (HFSS) are given in Figure 1, and the other parameters are: *L_s_* = 3.6 mm, *L_f_* = 15.8 mm, *L*_1_ = 4.2 mm, *L*_2_ = 10.7 mm.

### 2.2. Current Distributions of the Radiating Strips

**Figure 3:**
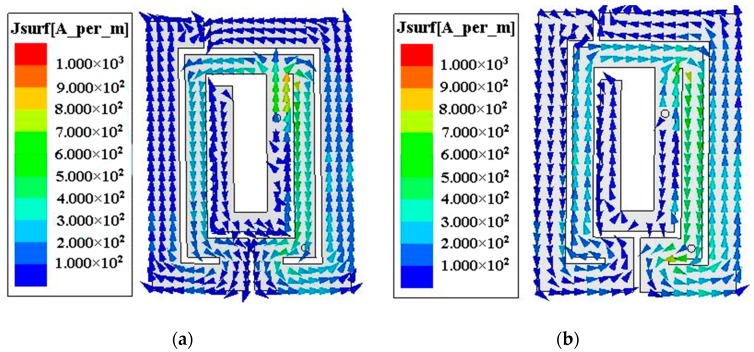
Current distributions of the proposed antenna at (**a**) 391 MHz; (**b**) 412 MHz.

In this work, the broad impedance bandwidth is due to a combination of two neighboring resonant frequencies at 391 MHz and 412 MHz. To better understand the operating mechanism, the current distributions of the proposed antenna at two resonant frequencies are shown in Figure 3. At 391 MHz, the current path of the inverted rectangular opening rings on the left side of the short via is opposite to one on the right side, and indicates that either part of the metal radiating strip resonates at this low frequency. At 412 MHz, the current of the two inverted rectangular opening rings flows in the same direction from one end to the other, and indicates that the whole metal strip contributes to the high resonant frequency. Thus, the antenna can realize dual resonances to obtain a broad impedance bandwidth. It is also worth mentioning that the current distribution at 402 MHz is similar to that at 412 MHz, which is not shown here for brevity.

### 2.3. Parametric Study

**Figure 4:**
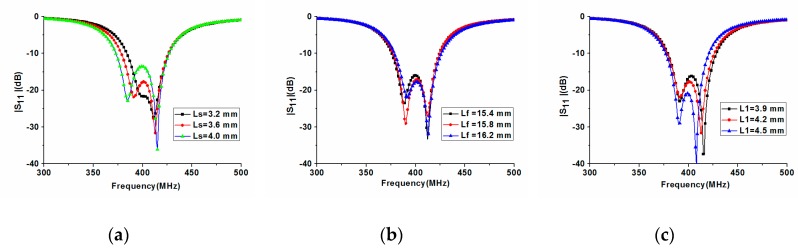
Comparison of |*S*_11_| of the proposed antenna with different geometric parameters: (**a**) short position *L_s_*; (**b**) feed position *L_f_*; and (**c**) tuned strip length *L*_1_.

In order to achieve the proper resonant frequencies in Figure 1, parameters *L_s_* (the short position), *L_f_* (the feed position), and *L*_1_ and *L*_2_ (the tuned strip length) are analyzed by the electromagnetic simulator Ansoft HFSS. To accurately assess the influence of these parameters, only one parameter at a time is varied while others are kept constant. Figure 4a shows that the shorter the length *L_s_*, the higher the low resonant frequency around 391 MHz. However, the short position *L_s_* has little effect on the high 412 MHz resonant frequency in the simulation. Therefore, we can conclude that the C-shaped strip in Figure 1 mainly contributes to the low 391 MHz resonant frequency. Figure 4b also shows the effect of the feed position *L_f_* on return loss. It is found that the longer the length *L_f_*, the higher the low resonant frequency around 391 MHz. Similarly, the feed position hardly affects the resonant frequency of 412 MHz.

In order to further analyze the operation principle of the antenna, an antenna with different tuned strip length *L*_1_ was examined. As shown in Figure 4c, the longer the length *L*_1_, the lower the high resonant frequency about 412 MHz. However, the tuned strip length *L*_1_ has almost no effect on the low 391 MHz resonant frequency. It is also worth pointing out that, according to simulation, the effect of *L*_2_ on resonant frequency is similar to that of *L*_1_. On the other hand, it can be concluded from Figure 3 that the total metal strip length contributes to the high resonant frequency, which is consistent with the conclusion of Figure 4c.

## 3. Results and Discussion

**Figure 5:**
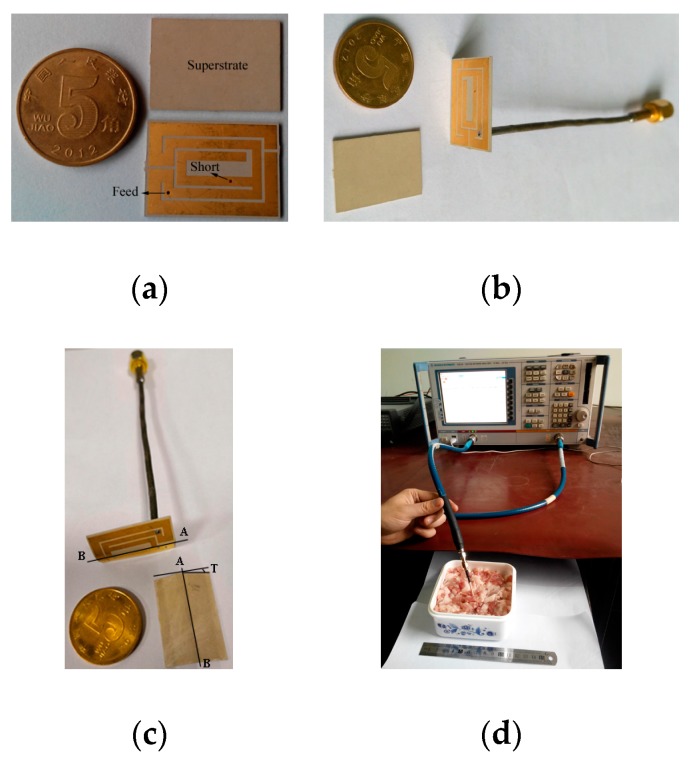
Photographs and experimental setup of the proposed antenna. (**a**) Photograph of the exploded flat antenna; (**b**) photograph of the assembled planar antenna; (**c**) photograph of the assembled bent antenna; (**d**) experimental setup.

**Figure 6:**
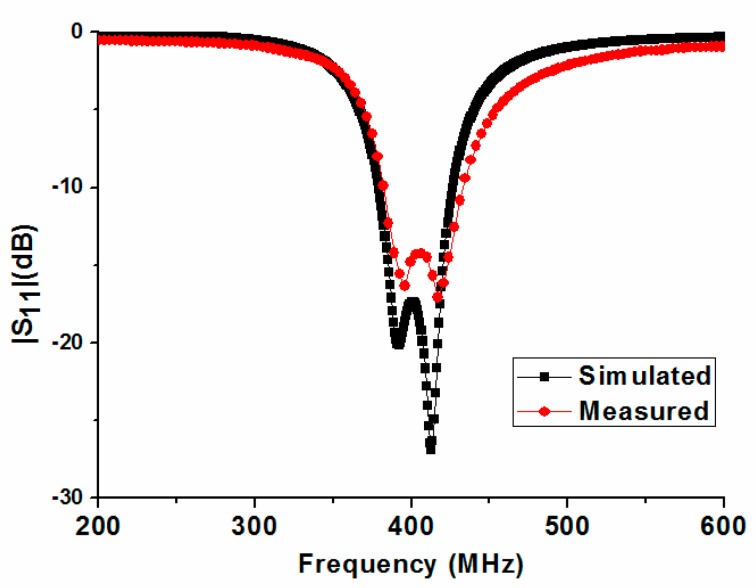
Measured and simulated |*S*_11_| of the proposed implantable antenna.

Photographs and the experimental setup of the fabricated antenna, including its superstrate, are shown in Figure 5. Figure 5a displays a photograph of the exploded flat antenna. The assembled flat and bent antennas with a 50 Ω coaxial cable are shown in Figure 5b,c, respectively. Figure 5d shows the experimental setup for the return loss measurement of the antenna in this study. According to [15], the permittivity and conductivity of chopped pork are similar to that of human skin in the MedRadio band, so the antenna was measured by using chopped pork. Figure 6 shows a comparison of measured and simulated return loss of the proposed antenna. The measured return loss agrees well with the simulation. The simulated −10 dB impedance bandwidth (IBW) is 49 MHz (378–427 MHz), and the measured |*S*_11_| is from 382 MHz to 434 MHz below −10 dB and has a bandwidth of 52 MHz, which completely covers the MedRadio band (401–406 MHz). Compared with the simulation, the slight frequency shift could be caused by unexpected fabrication tolerance and solder roughness.

**Figure 7:**
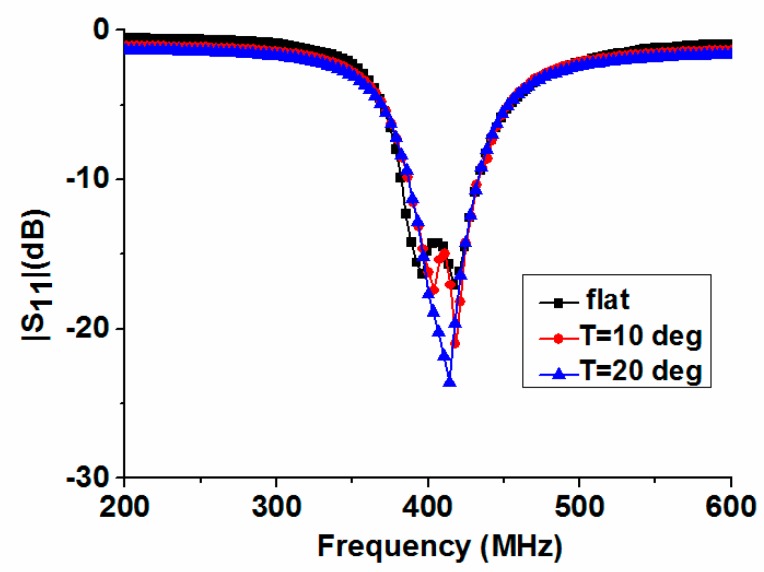
Measured |*S*_11_| of the flat and bent antenna with different angles.

Typically, the implanted antenna is placed in a fixed position in the stationary human body. When the human body moves, bent and twisted antennas can be used to mimic it. In order to further analyze the performance of the proposed antenna in different cases, we compared the measured |*S*_11_| of the flat case and two bent cases with different bent angles *T* = 10° and *T* = 20° along the line AB in Figure 5c. Here, the experimental setup of Figure 5d is still employed. Measured results of the flat and bent antennas are shown in Figure 7. When the antenna is bent along the line AB, the high resonant frequency of the antenna is slightly lowered, while the low resonant frequency significantly increases, so the −10 dB impedance bandwidth is continuously reduced. However, it continues to completely cover the MedRadio band. It should be noted that the antenna cannot be arbitrarily distorted due to the limitation of the selected substrate in this work. By using ultra-thin and well-stretched substrates, related experiments will be conducted in the future.

**Figure 8:**
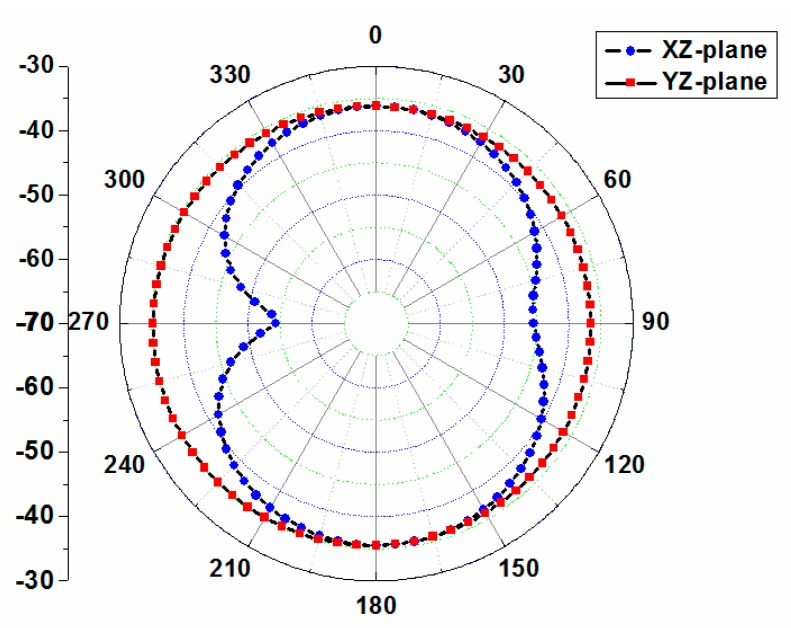
Simulated 2D patterns for the proposed implantable antenna at 402 MHz.

The simulated two-dimensional far-field gain pattern of the proposed antenna at 402 MHz is shown in Figure 8. The pattern is nearly omni-directional on the YZ-plane with a peak gain of −34.9 dBi. In addition, we have evaluated the 1-g averaged specific absorption rate (SAR) for consideration of human body safety concerns [16]. When the delivered power of the proposed antenna is assumed to be 1 W, the maximum SAR value at 402 MHz is 284.5 W/kg. Therefore, the allowed transmitter power is 5.62 mW to satisfy the 1-g SAR regulation.

The size, −10 dB impedance bandwidth and peak gain of the proposed antenna are compared with those of previous broadband implantable antennas in the MICS or MedRadio bands in Table 1. From Table 1, we can see that our antenna has a wider bandwidth than other compact narrowband antennas [6,7]. In [8,9], complex stacked structures were used. A broadband monopole antenna with C-shaped coupled ground was adopted in [10], and has a slightly larger volume. In our work, by exciting dual resonant frequencies, a new implantable antenna with a single-layer radiating patch was proposed. Therefore, compared to similar broadband antennas [8,9,10], our antenna has good comprehensive performance in terms of structure, volume and bandwidth. It can be concluded that our antenna has a new structure and good comprehensive performance based on the results of the comparison.

## 4. Conclusions

In this paper, we propose a broadband implantable antenna and discuss the bandwidth-increasing technology of implanted antennas. By using a bending and shorting strategy for a single-layer metal radiating patch, a novel dual-resonant implantable antenna in the MedRadio band has been presented. The proposed antenna has a 52-MHz measured bandwidth and a compact planar structure, without stacked metal radiating layers. The size of the proposed antenna is 23 × 16.4 × 1.27 mm^3^, which can be further reduced to meet the requirements of implantable microdevices by using a high dielectric constant and ultra-thin substrate. Good agreement is obtained between simulation and measurement for the return loss. In order to mimic human motion, the measured |*S*_11_| of the antenna in a flat and two bent cases is compared. Additionally, the radiation performance of the antenna and the human body safety performance were evaluated. The proposed antenna would be a promising candidate for implantable devices in the biotelemetry field owing to its advantages.

## Figures and Tables

**Table 1:** Performance comparison of the proposed antenna and other similar implantable antennas.

Reference	Volume (mm^3^)	−10 dB Impedance Bandwidth (IBW) (MHz)	Peak Gain (dBi)
[6]	198	394–419	−32.4
[7]	151	398–423	−33.2
[8]	190	385–425	−26.0
[9]	791	353–473	−27.2
[10]	560	368–687	−28.0
This paper	479	378–427	−34.9
